# Brachytherapy of stage II mobile tongue carcinoma. Prediction of local control and QOL

**DOI:** 10.1186/1748-717X-1-21

**Published:** 2006-07-12

**Authors:** Sayako Oota, Hitoshi Shibuya, Ryo-ichi Yoshimura, Hiroshi Watanabe, Masahiko Miura

**Affiliations:** 1 Department of Radiology, Asahi General Hospital, I-1326, Asahi, Chiba, Japan; 2 Department of Radiology, Tokyo Medical and Dental University, 1-5-45 Yushima, Bunkyo, Tokyo, Japan

## Abstract

**Background:**

There is no consensus as to the prognostic model for brachytherapy of tongue carcinoma. This study was designed to evaluate the prognostic factors for local control based on a large population under a unified treatment policy.

**Results:**

Between 1970 and 1998, 433 patients with stage II tongue squamous cell carcinoma were treated by low-dose-rate brachytherapy. This series included 277 patients treated with a linear source with a minimum follow-up of 3 years. A spacer was introduced in 1987. The primary local control rates were 85.6%.

**Conclusion:**

In the multivariate analysis, an invasive growth pattern was a significant factor for local recurrence. The disease-related survival was influenced by old age and an invasive growth pattern. A spacer lowered mandibular bone complications. The growth pattern was the most important factor for recurrence. Brachytherapy was associated with a high cure rate and the use of spacers brought about good quality of life (QOL).

## Background

Brachytherapy is frequently chosen for the treatment of stage II mobile tongue cancer, so as to avoid the large tissue defects caused by surgery, and conserve good function. Since surgery of T1 tumors is associated with good results in terms of the prognosis and function, the ratio of patients with T2 tumors who undergo brachytherapy has increased lately.

There is relatively little information in the literature on the prognostic factors within subgroups of patients undergoing brachytherapy for tongue cancer, and there is as yet no consensus as to the best prognostic model. Given the scarcity of adequate analyses using a consistent number of variables, new studies using large control groups, especially those deriving from a single institution and falling under the umbrella of a consistent treatment policy, are needed. We evaluated variables to determine their potential for predicting local control, survival and QOL, with the aim of providing a more effective post-treatment follow-up protocol. Leukoplakia is a white patch on the oral mucosa that can neither be scraped off nor classified as any other diagnosable disease (World Health Organization 1978) [1] and is known to frequently co-exist with tongue cancer, although there has been no study determining the correlation of its presence with the treatment result [2]. In addition, it was reported that in 74% of cases, local recurrence occurred within 2 years of treatment [3]. This study is based on data from local lesions without N factor at the first clinical consultation that could be observed for at least 3 years, and the duration of follow-up was longer than in previous studies, to allow understanding of the natural behavior of local lesions.

## Methods

433 patients with squamous cell carcinoma of stage II mobile tongue were treated with low dose rate brachytherapy at our institute for a 28-year period.

Between 1970 and 1998, 433 patients underwent treatment by brachytherapy for stage II squamous cell carcinoma of the tongue at the Tokyo Medical and Dental University Hospital. This series included 337 patients with a minimum follow-up of local disease for 3 years to reflect the nature of local disease. 277 patients were treated with linear ^192^Ir, ^226^Ra or ^137^Cs needle and 60 patients were treated with ^198^Au or ^226^Rn grain. Median follow-up was 7 years and 7 months for 337 patients and 7 years and 10 months for 277 patients treated by linear source. Statistical analysis was finally conducted from patients in whom observation of local disease could be obtained for at least 3 years, or who died of local recurrence within 3 years of therapy. 201 of these patients were male and 136 were female, and the median age of the patients was 56 years (range: 19–92 years). In this study, the tumors were categorized for analysis as 1) exophytic, referring to tumors showing an external growth 2) superficial, referring to flat tumors without palpable infiltration, and 3) invasive, referring to tumors penetrating deeply with or without ulceration, based on the findings of inspection and palpation. Tumor thickness was judged clinically and measured with vernier calipers.

All the patients were treated by low-dose rate brachytherapy with or without external irradiation. None of the patients received prophylactic neck irradiation. Small tumors less than 10 mm in tumor thickness were treated using a single-plane implant. Tumors between 10 mm and 15 mm in tumor thickness were treated using a single-plane implant with extra implant in cases where the dose distribution needed to be corrected. Tumors thicker than 15 mm were treated using a double-plane or volume implant. Leukoplakia adjacent to the tongue cancer was included within the treatment volume. The dose was calculated with the Manchester system. Pre-treatment estimation of treatment time was made by Paterson-Parker's table and 70 Gy was set as the reference dose. Since 1976, the actual dose distribution curve has been obtained by computer dosimetry using rectangular X-ray films taken about 24 hours after implantation. No tomographic image was obtained for calculation.

In some patients with a tumor diameter more than 30 mm or when brachytherapy cannot be performed immediately, 30 to 40 Gy of external irradiation or intra-oral cone electron beam therapy to the primary site was employed. The radiation field of external irradiation was shaped to give at least a 2 cm margin and the inferior part of the field usually lay at the thyroid notch. Brachytherapy was performed to give 60 Gy to the target volume in such cases.

Spacers made of translucent acrylic resin and ball clasps as a locking device have been used since 1987 and were used during the implantation [4]. The thickness of the lingual section was designed to obtain about 10 mm with a minimum of 7 mm.

Complication was evaluated clinically. After the treatment, the patients were seen approximately every 2 weeks for the first 3 months, every month during the first year, every 6 months during the next 2 years, and every year thereafter. When recent follow-up was not available, the surviving patients or their families were contacted for anamnestic search. Data was interpreted according to National cancer institute-Common terminology criteria for adverse events, version 3.0 for early complication and Radiation Therapy Oncology Group(RTOG)/European Organization for Research and Treatment of Cancer (EORTC) Late radiation morbidity scoring scheme for late complication. Biopsy was conducted in cases where the clinical findings were considered insufficient for diagnosis and in all cases where salvage surgery was performed. If a patient developed metastasis and subsequently presented with local failure, he/she was scored as having local failure. Death from local failure was defined death from local recurrence of cancer that could not be controlled and the tumor continued to grow. The survival rate was analyzed in the 277 patients treated using a linear source. The primary relapse-free survival (RFS) was calculated as the time from the first day of treatment to the date of the last follow-up. Disease-related survival (DRS) was calculated as the time from the date of the first visit to that of the last follow-up. In the case of disease-related survival, only deaths due to carcinoma of the tongue were counted, and patients who died of other causes were considered lost to follow-up. The overall median follow-up duration was 7 years and 10 months (minimum, 5 months; maximum, 29 years and 9 months). The survival patterns were estimated using the actuarial method. The difference of post-treatment condition between with or without spacer was analyzed using t-test and Fischer's direct method. The correlations among the various prognostic factors were assessed by the chi-square method. The survival data of the different subgroups of patients were compared using the Mantel-Haenszel log-rank test. A multivariate analysis was performed using the Cox proportional hazards model. The statistical analyses were performed using StatView software (SAS Institute, Cary NC).

## Results

Brachytherapy was performed with ^192^Ir hairpin in 104 patients (31%), ^226^Ra needle in 162 (48%), ^137^Cs needle in 11 (3%) and ^198^Au or ^222^Rn grain in 60 (18%). The total dose calculated from the reference isodose was 60–85 Gy (median 70 Gy) with 83 Gy of mean central dose in cases treated with ^192^Ir hairpin, ^226^Ra needle or ^137^Cs needle in five to seven days and 60–105 Gy (median 90 Gy) in seven days with ^198^Au or ^222^Rn grain. The 2 and 5 year relapse free rate was 88.5%, 85.4% (^192^Ir), 81.8%, 81.8% (^137^Cs), 90.7%, 87% (^226^Ra), 62.8%, 54.4% (^198^Au) and 64.7%, 58.8% (^222^Rn) and significantly worse in point sources (grains) compared to linear sources (needles) (p < 0.0001).

^198^Au and ^222^Rn seeds are small, and treatment does not require complex manoeuvres of self-care. These sources tend to be prescribed for patients who cannot be treated by other means because of complications or old age. This method is also associated with differences in the dose distribution, as grain implantation is more likely to make cold spots than needles [5]. In order to avoid bias, patients treated with the linear sources were analyzed separately excluding patients treated with the point sources. The characteristics of selected cases are listed in Table 1.

**Table 1:** Variables and Relative Classes for 277 patients treated with linear sources

**Variables**	**Classes**	**No.**	**%**
Sex	Male	169	61
	Female	108	39
Age at diagnosis (yrs)	≤60	174	63
	> 60	103	37
Growth pattern	Superficial	81	29
	Exophytic	121	44
	Invasive	75	27
Site	Side	254	92
	Tip	1	0
	Lower	20	7
	Upper	2	1
Leukoplakia	Absent	67	24
	Present	210	76
Tumor thickness (mm)	≤5	106	38
	6–10	97	35
	11–15	40	14
	> 15	34	12
Maximum diameter (mm)	≤30	196	71
	> 30	81	29
Brachytherapy source	192Ir hairpin	104	38
	226Ra needle	162	58
	137Cs needle	11	4
Brachytherapy dose (Gy)	≤70	216	78
	> 70	61	22
Brachytherapy plane	Single	242	87
	Double	32	12
	Volume	3	1
External irradiation	+	45	16
	-	232	84

The actuarial estimates were plotted (Figure 3). The relapse-free duration ranged from 2 to 357 months, with a mean and median survival of 8 years and 4 months and 7 years and 3 months. At two, three, five and 10 years, the probability of RFS was 89.5%, 87.4%, 86.2% and 85.0%, respectively. The two-, three-, five-, and 10-year DRS rates were 96.0%, 94.9%, 89.5% and 86.2%, respectively.

**Figure 1:**
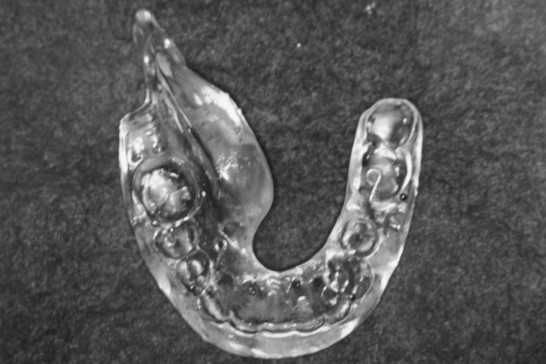
An acrylic resin spacer.

**Figure 2:**
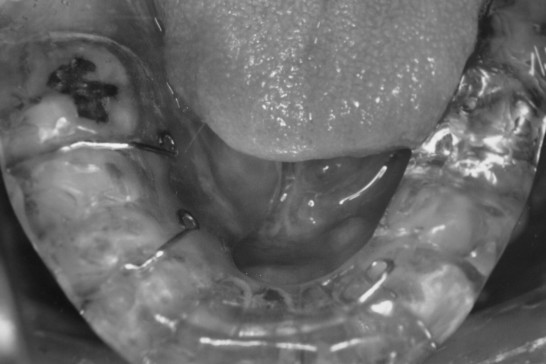
A spacer attached to the lower teeth.

**Figure 3:**
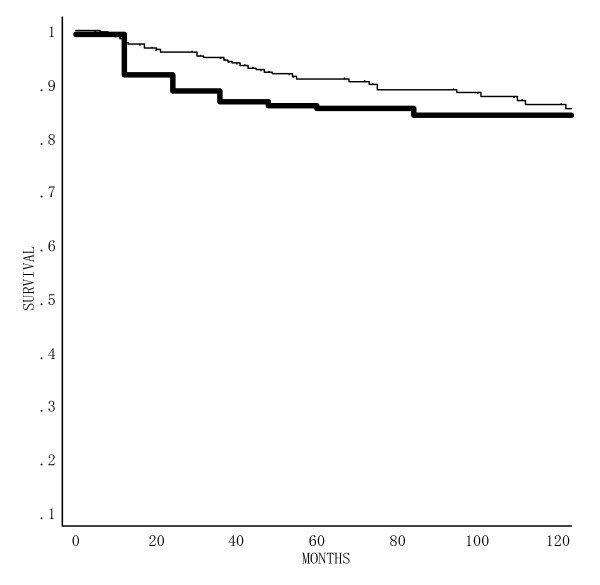
Survival curves for 277 patients with T2 tongue carcinoma treated with an ^192^Ir, ^137^Cs or ^226^Ra needle; Disease- related survival (); and relapse- free survival ().

**Figure 4:**
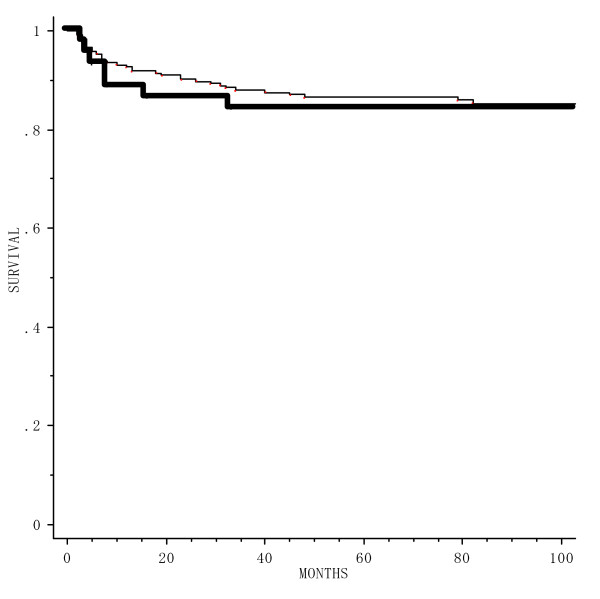
Relapse free survival for 277 patients treated with brachytherapy alone (); and with brachytherapy following external irradiation ().

45 patients underwent external irradiation prior to brachytherapy. The range of time interval between the first external irradiation day and implant was 14–70 days (median 34). ^60^Co external irradiation was used in 29 patients and 4 MV X-rays in 14. The total external radiation dose ranged from 10–45 Gy (median 26), with the daily fraction size varying from 2.0–2.5 Gy. Intra-oral cone electron beam therapy (4 MeV) was administered in 2 patients; the total dose were 10 and 40 Gy, with the total treatment time 5 and 46 days respectively. In these patients, the median brachytherapy dose was 60 Gy. At two and five years, the probability of RFS was 90.1% and 86.6% with brachytherapy only and 86.4% and 84.1% with external irradiation prior to brachytherapy (p = 0.75) (Figure 4).

**Figure 5:**
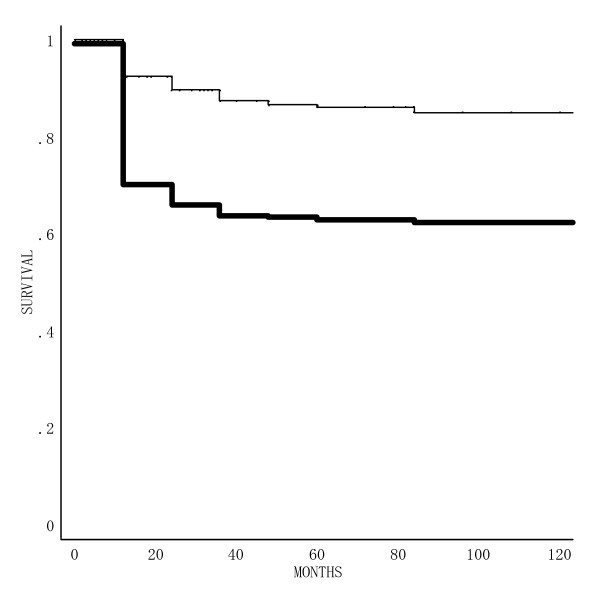
Survival curves for 277 patients with T2 tongue carcinoma treated with an ^192^Ir, ^137^Cs or ^226^Ra needle; Regional disease-free survival (); and distant metastasis-free survival ().

76 patients (27%) relapsed or had an event that removed them from the disease-free category. Those events were distributed as follows; local failure only, 15 patients (5%); regional failure only, 41 patients (15%); distant failure only, 2 patients (1%); and local plus regional and/or distant failure, 40 patients (14%). The failure pattern was further analyzed to assess specific 5-year cumulative survival rates according to the sites of relapse as follows; regional, 63.7% and distant 97.6% (Figure 5).

At the last follow-up, 207 patients were still alive without disease. 70 were dead, of which 29 died of their disease (Table 2); of these 29, 15 died within 3 years of the diagnosis, 12 between 3 to 6 years of the diagnosis, and 2 between 6 to 8 years of the diagnosis. Three of these patients received salvage brachytherapy with ^198^Au seeds. The dose of the ^198^Au seeds was 61–73 Gy (median 66 Gy) administered over seven days. Salvage external irradiation was given with ^60^Co in 4 cases. The total dose of external irradiation by ^60^Co was 12.5–50.2 Gy (median 20 Gy), with the daily dose varying between 2.0–2.5 Gy. A 10 MeV electron beam was prescribed in one case, with a total dose 46 Gy and the dose per day of 2 Gy. Intra-oral cone electron beam therapy was administered in 4 cases; the total dose was 29–30 Gy (median 30 Gy) and the total treatment time ranged from 8 to 15 days (mean 13). 26 patients underwent surgery only. No further aggressive management was attempted in 10 patients. Twenty-one patients were salvaged by surgery, 3 by brachytherapy alone, 4 by intra-oral irradiation, and 1 by external irradiation. None of the patients who received no further treatment could be salvaged. The overall final primary control rate was 95%. Table 3 shows the results of the univariate analysis of RFS and DRS for 8 variables. There were no significant differences in the sex distribution between those showing RFS and DRS. There was no significant difference in the age of the patients showing RFS or DRS. The growth pattern was the superficial type in 81 patients (29%), the exophytic type in 121 patients (44%), and the invasive type in 75 patients (27%). Patients with the exophytic and invasive type of tumor growth carried a 2.8- and 3.9 -fold increased risk of local recurrence, and a 5.1- and 7.5- fold increased risk of death as compared to those with the superficial type of tumor growth. Leukoplakia around the tumor was present in 67 patients (24%) and absent in 210 (76%). Its presence did not influence the RFS or DRS rates. The tumor thickness was ≤5 mm in 106 (38%) patients, 5–10 mm in 97 (35%), 10–15 mm in 40 (14%) and > 15 mm in 34 (12%) patients. Those with a tumor thickness measuring 10–15 mm had a 3-fold risk of local recurrence as compared to those with a tumor thickness of < 5 mm. Patients with a tumor thickness measuring 10–15 mm or >10 mm had a 3.0- and 3.5-fold greater risk of local recurrence as compared to those with a tumor thickness of ≤5 mm. Patients with a tumor thickness measuring > 15 mm had a 3.9-fold greater risk of death as compared to those with a tumor thickness of = 5 mm. The maximum tumor diameter was ≤3 cm in 81 patients (29%) and > 3 cm in 196 patients (71%); this variable was not found to affect the RFS or DRS rates. The difference of brachytherapy source, ^192^Ir hairpin, ^137^Cs needle or ^226^Ra needle, was not found to influence the risk of local recurrence or death. The median brachytherapy dose was 70 Gy; the dose was < 70 Gy in 216 patients (78%) and > 70 Gy in 61 patients (22%). No significant difference in the risk of local recurrence or death was detected between these two groups.

The multivariate analysis for local recurrence revealed the invasive growth pattern of the tumor to be a significant risk factor. For overall survival, old age and the invasive growth pattern of the tumor were found to be the most important prognostic factors. The invasive growth pattern and tumor thickness were revealed to be significant factors for cervical node metastasis (Table 4).

**Table 2:** Patients' Current Status

**Status**	**Frequency (277 patients)**
Alive, with no evidence of disease	207
Alive with clinical evidence of disease	0
Dead of disease	29
Dead of other causes	41

**Table 3:** Univariate Analysis; significant factors

		**Relapse free survival**		**Overall survival**		**Cervical metastasis free survival**	
**Variables**		**UHR**	**P value**	**UHR**	**P value**	**UHR**	**P value**

Growth pattern							
	Superficial vs. exophytic	2.84	<0.001	5.16	0.03	1.49	0.15
	Superficial vs. invasive	3.92	<0.001	7.54	0.01	3.39	<0.001
Tumor thickness (mm)							
	≤5 vs. 6–10	1.76	0.19	2.08	0.15	2.87	<0.001
	≤5 vs. 10–15	3.02	0.02	2.80	0.08	3.10	<0.001
	≤5 vs. > 15	3.57	<0.001	3.93	0.01	5.02	<0.001
Brachytherapy source							
	192Ir vs. 137Cs	1.25	0.76	1.29	0.81	3.74	<0.001
	192Ir vs. 226Ra	0.81	0.52	1.22	0.61	1.23	0.33
Brachytherapy dose (Gy)							
	≤70vs. > 70	1.24	0.54	1.24	0.61	1.03	0.91
UHR: unadjusted hazard ratio							

**Table 4:** Multivariate Analysis; significant factors

		**Relapse free survival**		**Overall survival**		**Cervical metastasis free survival**	
**Variables**		**AHR**	**P value**	**AHR**	**P value**	**AHR**	**P value**

Growth pattern							
	Superficial vs. exophytic	2.17	0.84	4.93	0.06	0.70	0.15
	Superficial vs. invasive	3.15	0.02	7.69	0.02	1.26	<0.001
Tumor thickness (mm)							
	≤5 vs. 6–10	0.82	0.62	1.02	0.98	2.85	<0.001
	≤5 vs. 10–15	2.10	0.09	1.25	0.73	3.02	<0.001
	≤5 vs. > 15	1.24	0.66	1.93	0.33	5.47	<0.001
AHR: adjusted hazard ratio							

Grade 1–2 mucositis was seen in all the patient as early complication and no patient showed toxicity greater than Grade 3. Mucositis was ameliorated with median period of 3 months. Prolonged complication more than 6 months was seen in 26 patients (9%). 6 of them had a problem of compression by adjacent teeth. QOL of the patients became remarkably better after the introduction of a spacer between the tongue and the mandible (Fig 1 and 2). Formerly, the incidence of Grade 4 mandibular complication was high (22% = 23/105) in the cases without local recurrence and 8 cases required operation. After the use of spacers began, the incidence of mandibular complication reduced (6% = 7/119), and no salvage operation was performed. In addition, salvage operation for Grade 4 mucositis was necessitated in 3 cases of 103 patients treated without a spacer, while no operation was required by patients treated with a spacer. The interval of radiation mucositis after implantation was longer in the non-spacer group (110 days) compared to the spacer group (84 days), although it was not significant (p = 0.07). After the introduction of computer dosimetry, local ulcers caused by overdose have decreased to zero.

Second tumors occurred in 73 patients and in 53 of them primary tumors and regional metastases were clinically controlled.

Some cases showed co-existing cancers including 20 oral cancers, 15 esophageal cancers, 11 lung cancers, 8 oro-hypopharyngeal cancers, 5 stomach cancers and another 8 tumors in the treated area with long latent periods over 8 years.

## Discussion

The 5-year local control rate determined in the current patient series was not significantly different from the figures published during the last 2 decades [6-11], although it was slightly higher [12]. The current study, based on the largest series so far, whose protocol included a relatively long follow-up period and consisted of subjects that fell under a unified treatment policy, was performed with the aim of drawing conclusions on the natural history of the disease and the post-treatment condition.

It was previously reported that the growth pattern of the carcinoma did not have a statistically significant effect on the disease-specific survival, local recurrence or overall survival of the patients [13]. In an early report by Paterson, the tumor growth type was categorized into four types; 1) papillary or fungating, 2) superficial, 3) ulcerative and 4) infiltrating [14]. It was clinically difficult to distinguish the ulcerative type from the infiltrating type by appearance because they frequently grow into deep tissue invasively. It has been suggested that the ulcerative or infiltrating types of tumors are difficult to treat, because of their tendency to invade deeply into adjacent tissue. In the current study, the results appeared to support the impression that the invasive growth type affects the frequency of local recurrence and survival unfavorably.

In contrast to the recent results reported by some groups, the tumor thickness was not found to be a predictive factor for local recurrence or survival in the final model [[13] and [15]]. The treatment policy of prescribing extra implants for thick tumors may have influenced the result. In addition, it is also possible that the tumor thickness did not exert any significant influence in our series, because exophytic tumors, with relatively better prognosis, and invasive tumors, with a worse prognosis, were analyzed together. However, further analysis was performed for both the growth patterns, and the results showed no significant influence of tumor thickness on the frequency of local recurrence or survival in either group.

Age has been both claimed and denied as a predictor of the prognosis [15-17]. In the current study, old patients were found to be at the risk of dying prematurely, although no significant influence of this parameter was seen on the frequency of local recurrence.

Leukoplakia is known as a premalignant or potentially malignant lesion of the oral mucosa [[2,9] and [18]]. Mucosal carcinomas associated with leukoplakia appear to be only superficially invasive and carry a better prognosis than similar carcinomas not associated with leukoplakia. In the current study, leukoplakia at the periphery of the tongue carcinoma was included in the target area of treatment, as a not significant predictive factor.

The tumor size or the T factor has been evaluated in previous studies and been shown to be an important predictor of local control [19,20]. In the current study, however, consistent with some previous reports, it was not found to affect survival or local control [15]. Since only patients with T2 disease were included in the analysis, the tumor diameter range was more limited than that in studies analyzing different T factors.

The higher death rate in cases with exophytic and invasive tumors was assumed to be a reflection of the associated increased rate of local recurrence. Nakagawa et al. reported that the invasive growth pattern was related to the risk of neck node metastasis and a higher death rate[21].

Contribution of external irradiation prior to brachytherapy thought to be small and brachytherapy dose should not be reduced.

In conclusion, the invasive growth pattern was found to be a strong predictive factor of local recurrence. Most of the other variables investigated in this study did not have any prognostic implications. Attempts have also been made to define predictive factors from the aspect of histopathology [[13,21] and [22]]. In addition, computer dosimetry and spacer use are indispensable procedures. However, QOL of oral cancer patients mainly depends on mucous and mandibular status, proper measurement using the EORTC QLQ-C30 questionnaire provides clear evaluation from various aspects. [23] A recent study revealed that high dose rate brachytherapy has advantage to concentrate high dose to the target and reduce the risk of normal tissue injury by optimization using tomographic anatomical information [[24] and [25]]. Modern technique such as 3D optimised target oriented dose application can also be applied to improve low dose rate brachytherapy.

## Competing interests

The author(s) declare that they have no competing interests.

## References

[B1] WHO collaborating centre for oral precancerous lesions (1978). Definition of leukoplakia and related lesions: an aid to studies on oral precancer. Oral Surg Oral Med Oral Pathol.

[B2] Waar van der I, Schepman KP, Meij van der EH (1997). Oral leukoplakia: a clinicopathological review. Oral Oncology.

[B3] Shibuya H, Hoshina M, Takeda M (1993). Brachytherapy for stage I & II oral tongue cancer: an analysis of past cases focusing on control and complications. Int J Radiat Oncol Biol Phys.

[B4] Miura M, Takeda M, Sasaki T (1998). Factors affecting mandibular complications in low dose rate brachytherapy for oral tongue carcinoma with special reference to spacer. Int J Radiat Oncol Biol Phys.

[B5] Delclos L, Lindberg RD, Fletcher GH (1976). Squamous cell carcinoma of the oral tongue and floor of mouth: Evaluation of interstitial radium therapy. Am J Roentgenol.

[B6] Lefebvre JL, Coche-Dequeant B, Castelain B (1990). Interstitial brachytherapy and early tongue squamous cell carcinoma management. Head Neck.

[B7] Leung TW, Wong VY, Kwan KH (2002). High dose rate brachytherapy for early stage oral tongue cancer. Head Neck.

[B8] Mazeron JJ, Crook JM, Marinello G (1990). Prognostic factors of local outcome for T1, T2 carcinomas treated by iridium 192 implantation. Int J Radiat Oncol Biol Phys.

[B9] Shibuya H, Amagasa T, Seto K (1986). Leukoplakia-associated multiple carcinomas in patients with tongue carcinoma. Cancer.

[B10] Bourgier C, Coche-Dequeant B, Fournier C (2005). Exclusive low-dose rate brachytherapy in 279 patients with T2N0 mobile tongue carcinoma. Int J Radiat Oncol Biol Phys.

[B11] Pourel N, Peiffert D, Lartigau E (2002). Quality of life in long-term survivors of oropharynx carcinoma. Int J Radiat Oncol Biol Phys.

[B12] Wendt CD, Peters LJ, Delclos L (1990). Primary radiotherapy in the treatment of stage I and II oral tongue cancers: importance of the proportion of therapy delivered with interstitial therapy. Int J Radiat Oncol Biol Phys.

[B13] Yuen APW, Lam KY, Lam LK (2002). Prognostic factors of clinically stage I and II oral tongue carcinoma-a comparative study of stage, thickness, shape, growth pattern, invasive front malignancy grading, Martinez-Gimeno score, and pathologic features. Head Neck.

[B14] Paterson R, Paterson R, Stewart JG (1963). The treatment of malignant disease by radiotherapy.

[B15] Al-Rajhi N, Khafaga Y, El-Husseiny J (2000). Early stage carcinoma of oral tongue: prognostic factors for local control and survival. Oral Oncology.

[B16] Siegelmann-Danieli N, Hanlon A, Ridge JA (1998). Oral tongue cancer in patients less than 45 years old: institutional experience and comparison with older patients. J Clin Oncol.

[B17] Yoshida K, Koizumi M, Inoue T (1999). Radiotherapy of early tongue cancer in patients less than 40 years old. Int J Radiat Oncol Biol Phys.

[B18] Sudbø J, Bankfalvi A, Bryne M (2000). Prognostic value of graph theory-based tissue architecture analysis in carcinomas of the tongue. Lab Invest.

[B19] Hoşai AŞ, Ünal ÖF, Ayhan A (1998). Possible prognostic value of histopathologic parameters in patients with carcinoma of the oral tongue. Eur Arc Otorhinolaryngol.

[B20] Nyman J, Mercke C, Lindström J (1993). Prognostic factors for local control and survival of cancer of the oral tongue A retrospective analysis of 230 cases in Western Sweden. Acta oncologica.

[B21] Nakagawa T, Shibuya H, Yoshimura R (2003). Neck node metastasis after successful brachytherapy for early stage tongue carcinoma. Radiother Oncol.

[B22] Silverman S, Gorsky M, Lozada F (1984). Oral leukoplakia and malignant transformation. Cancer.

[B23] Pernot M, Malissard L, Hoffstetter S (1994). The study of tumoral, radiobiological, and general health factors that influence results and complications in a seriese of 448 oral tongue carcinomas treated exclusively by irradiation. Int J Radiat Oncol Biol Phys.

[B24] Kolotas C, Baltas D, Zamboglou N (1999). CT-Based interstitial HDR brachytherapy. Strahlenther Onkol.

[B25] Umeda M, Komatsubara H, Nishimatsu N (2000). High-dose rate interstitial brachytherapy for stage I-II tongue cancer. Oral Surg Oral Med Oral Pathol Oral Radiol Endod.

